# *Ex Vivo* Stretch Reveals Altered Mechanical Properties of Isolated Dystrophin-Deficient Hearts

**DOI:** 10.1371/journal.pone.0032880

**Published:** 2012-03-09

**Authors:** Matthew S. Barnabei, Joseph M. Metzger

**Affiliations:** Department of Integrative Biology and Physiology, University of Minnesota Medical School, Minneapolis, Minnesota, United States of America; Medical College of Georgia, United States of America

## Abstract

Duchenne muscular dystrophy (DMD) is a progressive and fatal disease of muscle wasting caused by loss of the cytoskeletal protein dystrophin. In the heart, DMD results in progressive cardiomyopathy and dilation of the left ventricle through mechanisms that are not fully understood. Previous reports have shown that loss of dystrophin causes sarcolemmal instability and reduced mechanical compliance of isolated cardiac myocytes. To expand upon these findings, here we have subjected the left ventricles of dystrophin-deficient mdx hearts to mechanical stretch. Unexpectedly, isolated mdx hearts showed increased left ventricular (LV) compliance compared to controls during stretch as LV volume was increased above normal end diastolic volume. During LV chamber distention, sarcomere lengths increased similarly in mdx and WT hearts despite greater excursions in volume of mdx hearts. This suggests that the mechanical properties of the intact heart cannot be modeled as a simple extrapolation of findings in single cardiac myocytes. To explain these findings, a model is proposed in which disruption of the dystrophin-glycoprotein complex perturbs cell-extracellular matrix contacts and promotes the apparent slippage of myocytes past each other during LV distension. In comparison, similar increases in LV compliance were obtained in isolated hearts from β-sarcoglycan-null and laminin-α_2_ mutant mice, but not in dysferlin-null mice, suggesting that increased whole-organ compliance in mdx mice is a specific effect of disrupted cell-extracellular matrix contacts and not a general consequence of cardiomyopathy via membrane defect processes. Collectively, these findings suggest a novel and cell-death independent mechanism for the progressive pathological LV dilation that occurs in DMD.

## Introduction

Duchenne muscular dystrophy (DMD) is an inherited disease of progressive muscle wasting and is the most common form of muscular dystrophy, affecting 1 in 3500 males [1]. The DMD gene resides on the short arm of the X chromosome and encodes the protein dystrophin [2], [3], [4]. Dystrophin is a 427 kDa cytoskeletal protein and is a member of the dystrophin-glycoprotein complex (DGC) in striated muscles [2], [5], [6]. The DGC is a multimeric protein assembly that spans the sarcolemma, tethering the extracellular matrix to the cytoskeletal architecture of the cell [7]. Dystrophin is a cytoplasmic component of the DGC and plays a crucial role in this trans-sarcolemma linkage, binding cytoplasmic γ-actin at its N-terminus and the membrane glycoprotein β-dystroglycan at its C-terminus [8], [9], [10]. The DGC protein α-dystroglycan interacts with the extracellular matrix by binding laminin in a glycosylation dependent manner [7], [11]. In the absence of dystrophin, the mechanical rigors of muscle contraction are damaging to the sarcolemma. This mechanical instability has been observed in dystrophin-deficient skeletal muscle, which is highly susceptible to lengthening contraction-induced sarcolemmal damage and necrotic cell death [12], [13]. Previously, we and others have demonstrated similar mechanical instability in isolated single cardiac myocytes, by showing that loss of dystrophin causes the formation of “micro-tears” in the sarcolemma during passive mechanical distension of myocyte [14], [15], [16]. Recently, loss of dystrophin has also been shown to increase susceptibility to mechanical injury in multicellular cardiac trabecular preparations [17]. These sarcolemmal disruptions result in increased single-cell stiffness with eventual hypercontracture and cell death during physiological stretch due to unregulated influx of extracellular calcium [15], [16].

Clinically, DMD is characterized by skeletal muscle weakness and wasting. DMD presents early in life and is rapidly progressive, resulting in loss of ambulation at approximately 10 years of age and premature death in the early twenties [18], [19], [20], [21], [22]. Progressive cardiac dysfunction is a significant component of DMD which has been observed since the very first descriptions of the disease [21], [23]. Subclinical cardiac involvement is present in the majority of DMD patients in the first decade, the severity of which may be masked by significant skeletal muscle dysfunction [21], [24], [25]. In the later stages of DMD, nearly all patients show clinically significant cardiac muscle disease [26], [27]. Structurally, the cardiomyopathy of DMD is characterized by fibrosis and progressive dilation of the left ventricle (LV), leading to dilated cardiomyopathy (DCM) [27], [28]. This frequently progresses to heart failure, which is the primary cause of death in at least 20% of DMD patients [21], [26].

There are significant gaps in knowledge regarding the progression of cardiac involvement in DMD. Increased myocyte passive stiffness secondary to membrane damage in dystrophic myocytes may cause remodeling of LV dimensions early in DMD [15]. At present, how dystrophin deficiency causes LV dilation as the disease progresses is unclear. Previous findings regarding the progression of DCM in the non-dystrophic heart suggest that progressive LV dilation may involve myocyte loss, fibrosis, hypertrophy, and myocyte slippage [29], [30], [31], [32], [33], [34], [35]. The degree to which these processes contribute to cardiac dysfunction and dilation in DMD is currently unknown.

In order to build on previous findings in single cardiac myocytes and to gain new mechanistic understanding of the effects of mechanical stress on the dystrophic heart, we tested the hypothesis that isolated dystrophin-deficient hearts have reduced organ level compliance and increased susceptibility to mechanical damage. Using a modified *ex vivo* isolated heart preparation, results show, unexpectedly, that isolated mdx hearts have increased whole-organ compliance compared to normal hearts and tolerate considerable LV chamber distension without showing evidence of myocyte damage. To provide further mechanistic insight, we tested other genetic models of dystrophy in which either the DGC or native membrane repair apparatus were disrupted. We found that defective organ level compliance was evident only when the DGC or its extracellular binding partner laminin were disrupted but not in hearts lacking the membrane repair protein dysferlin.

Collectively, these results provide evidence that disruption of the DGC predisposes the LV to dilation via apparent myocyte slippage, a novel and cell death-independent mechanism for the progressive LV dilation observed in DMD.

## Results

**Figure 1:**
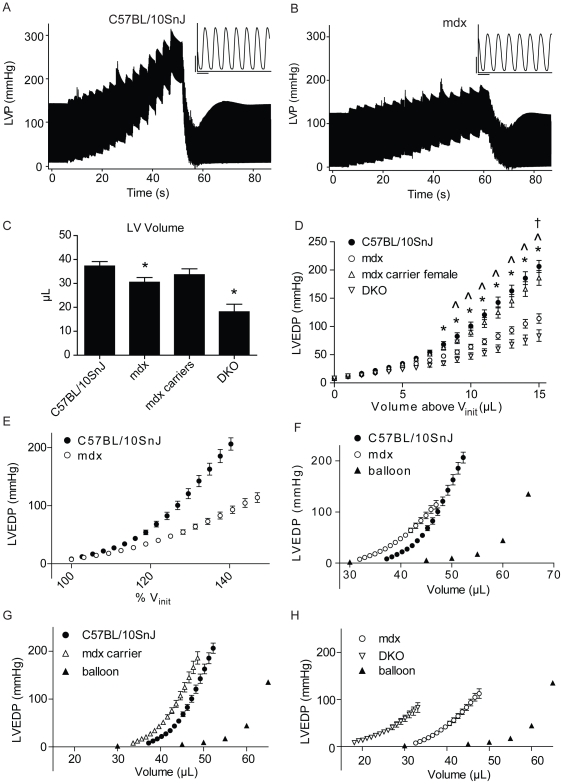
Response of isolated, contracting dystrophic isolated hearts to *ex vivo* stretch. A, B) Compressed representative pressure tracings from WT(A) and mdx (B) hearts during *ex vivo* stretch protocol. Inset: uncompressed pressure tracings from mdx and WT hearts prior to stretch, vertical scale bar = 50 mmHg, horizontal scale bar = 1/7 sec. C) Volume required to reach 8 mmHg EDP within the LV (V_init_). D) Altered whole-organ compliance of mdx and DKO hearts compared to WT as shown by plotting LVEDP against volume added above V_init_ during *ex vivo* stretch. Mdx carrier females show normal whole-organ compliance. E) Differences in compliance of mdx and WT hearts are further exaggerated when plotting %V_init_ versus LVEDP. F) Estimation of the true LV volume-EDP relationship based on data from 1C and 1D. Balloon only controls are shown to prove that recorded changes in pressure were not due to overfilled balloon. G) Whole-organ compliance of isolated WT and mdx carrier female hearts shown as the LV volume versus LVEDP. H) Whole-organ compliance of isolated mdx and DKO hearts shown as LV volume versus LVEDP. n = 5–14. For C, * - P<0.05 by t-test. For D, * - p<0.05 for C57BL/10SnJ vs. DKO, ∧ - P<0.05 for C57BL/10SnJ vs. mdx, † - p<0.05 for mdx versus DKO by two-way ANOVA with main effects for strain, volume, and an interaction of the two. Values expressed as mean ± SEM.

Previously, we have shown that the sarcolemma of single, membrane intact, mdx cardiac myocytes is highly susceptible to passive stretch-induced injury leading to increased stiffness, membrane permeability, aberrant extracellular calcium influx, and myocyte death [15]. To expand upon these findings and determine how single-cell mechanics manifest at the whole-organ level, the effects of mechanical stretch on the LV chamber of WT and mdx hearts were determined (Figure 1).

### Mechanical compliance of intact, beating mdx hearts

To determine whole-organ cardiac compliance, wild-type (WT, C57BL/10SnJ) and mdx hearts were isolated, perfused, and subjected to mechanical stretch using a water filled plastic balloon placed within the LV chamber. WT and mdx mice used in this study were of similar age (65±0.9 vs. 67±1.8 days, n = 14–16, P>0.05), had similar body weights (27.1±0.4 vs. 27.4±0.9 grams, n = 14–16, P>0.05) and heart weights (176.4 mg±5.4 vs. 158.8±10.3 milligrams, n = 5, P>0.05). Miniature plastic balloons were first inflated and set at standard physiological LV end diastolic pressure (LVEDP) of 6–8 mmHg [15], [36]. Baseline LV hemodynamic performance of mdx and WT hearts was similar (Table S1). In order to estimate the LV volume of isolated hearts at standard LVEDP, the volume required to reach LVEDP of 8 mmHg was recorded (V_init_, Figure 1c). In agreement with previously published findings, we found that mdx hearts have reduced LV volumes at normal LVEDP [15]. To assess whole-organ compliance in isolated hearts, 15 µL was added to the balloon in the LV chamber in 1 µL increments while simultaneously recording LV pressure. Unexpectedly, as volume was added above V_init_ mdx hearts showed a blunted increase in LVEDP, indicating an increase in whole-organ compliance compared to WT hearts (representative tracings shown in Figure 1a, b). This blunted increase in LVEDP as LV volume increased above V_init_ is quantified in Figure 1d, showing significant main effects of strain, volume and an interaction of the two. To account for differences in V_init_ between WT and mdx hearts, changes in LVEDP during stretch were also plotted against %V_init_. Similar results were observed when plotting the results this way despite the smaller V_init_ of mdx hearts, further accentuating the increased whole-organ compliance of mdx hearts (Figure 1e). To visualize the true LV EDP-volume relationship, LVEDP was plotted against LV volume (V_init_+volume added during stretch protocol, Figure 1f). Here, mdx hearts initially showed a left-shifted LVEDP-volume curve compared to WT at lower volumes. As LV volume was increased, the blunted slope of the LVEDP-LV volume relationship in mdx hearts approached that of WT hearts (Figure 1f). Collectively, these results demonstrate the increased compliance of isolated mdx hearts compared to WT.

Because we have previously shown that LV dimensions are normalized in mdx mice upon application of membrane sealant poloxamer 188 *in vivo* [15], we conducted parallel studies in isolated hearts perfused with buffer containing P188. Inclusion of P188 did not affect the increase in LVEDP as volume was added above V_init_ in isolated mdx hearts, suggesting that the alteration in mechanical compliance we observe are not related to mechanical instability of the sarcolemma (Figure S1a, b).

To determine if the observed differences in whole-organ compliance were due to mechanical injury of the heart or to ischemia caused by high LV pressures, we recorded the flow rate of perfusate through the coronary vasculature. Throughout the *ex vivo* stretch protocol there was no difference in coronary flow between mdx and WT hearts (Figure S1c). Additionally, to determine if the observed differences in whole-organ compliance were due to mechanical injury of mdx hearts during the stretch protocol, we assessed lactate dehydrogenase (LDH) activity in perfusates from isolated hearts before and after stretch. WT and mdx hearts showed similar initial perfusate LDH activity and showed no increase in perfusate LDH activity following stretch (Figure S1d). All values for LDH activity recorded for hearts used in the stretch protocol were less than those recorded following ischemia/reperfusion injury, suggesting that minimal cell injury or death occurred during the stretch protocol. Additionally, isolated hearts recovered cardiac performance to pre-stretch levels following the *ex vivo* stretch protocol (Figure 1a, b and Figure S1e). Collectively, these findings are evidence that the stretch protocol used in this study causes minimal injury to isolated hearts and had negligible effects on post-stretch protocol cardiac function.

To further characterize the effects of altered dystrophin expression on the mechanical properties of the myocardium, isolated hearts of mdx carrier females were subjected to the LV chamber stretch protocol. In these mice, 50% of cardiac myocytes lack dystrophin due to random X inactivation [37]. Isolated hearts of mdx carrier females showed whole-organ compliance similar to WT hearts (Figure 1d, g). We also assessed the whole-organ compliance of hearts from the dystrophin/utrophin double knockout (DKO) mouse. Utrophin is a homolog of dystrophin which is upregulated in mdx mice, compensating for dystrophin loss and contributing to a relatively mild phenotype compared to DMD patients [38], [39], [40]. DKO mice have a severe dystrophic phenotype which is similar to DMD patients [38], [39], [40]. DKO mouse hearts showed increased compliance compared to both WT and mdx (Figure 1d, h). DKO mice were studied at 4 weeks of age due to their severe dystrophic phenotype and had smaller LV volumes (Figure 1c). Because similar volumes were introduced to all hearts regardless of V_init_, smaller hearts would be predicted to show greater increases in LVEDP as LV volume is increased V_init_ if compliance were equivalent. Whole-heart compliance was reduced in DKO mice compared to controls despite this smaller initial volume. The sarcomeric protein titin is a central determinant of passive compliance in cardiac muscle [41], [42]. Alternative splicing of titin early in life may contribute to altered cardiac compliance as the heart develops [43]. However, in the mouse, the adult isoforms of titin are predominant after day 5, suggesting alterations in titin isoform expression did not contribute to enhanced compliance in DKO hearts [43].

### Effects of altering perfusate calcium on mechanical properties of isolated dystrophic and WT hearts

**Figure 2:**
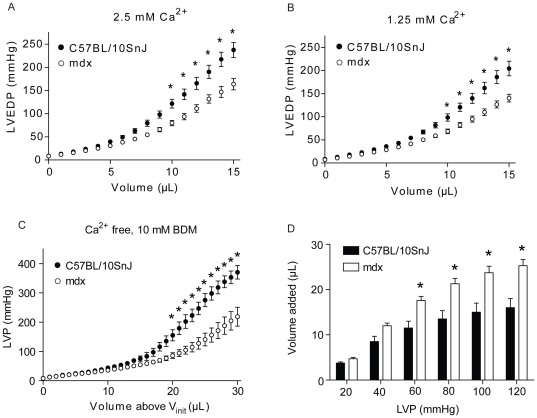
Response of non-contracting WT and mdx hearts to altered perfusate calcium. A, B) Altered compliance in mdx hearts as shown by plotting LVEDP against volume added above V_init_ during *ex vivo* stretch when perfused with 2.5 mM (A) or 1.25 mM (B) Ca^2+^. C) Increased compliance in non-contracting hearts perfused with BDM and without calcium during stretch as shown by plotting LVEDP against volume added above V_init_. D) Quantification of altered compliance in mdx hearts, plotted as volume required to raise LVP from 8 mmHg (V_init_) to 20, 40, 60, 80, 100, or 120 mmHg. n = 5–10. * - p<0.05 by two-way ANOVA with main effects for strain, volume, and an interaction of the two. Values expressed as mean ± SEM.

To determine the effects of altering extracellular calcium concentrations on the mechanical properties of the myocardium, isolated hearts were perfused with a modified Kreb's buffer containing high (3 mM) calcium or low (1.75 mM) calcium (all solutions contained 0.5 mM EDTA). Similar to our findings under standard conditions (2.5 mM calcium+0.5 mM EDTA) isolated mdx hearts perfused with high and low calcium showed a similar blunted increase in LVEDP as volume was increased above V_init_ as compared to controls (Figure 2a, b). To further characterize the effects of altering extracellular calcium on myocardial compliance, hearts were perfused with buffer lacking calcium and containing 10 mM 2, 3-butanedione monoxime to inhibit excitation-contraction coupling. Perfusion with this solution halted all contractile activity of isolated hearts. Non-contracting hearts were then subjected to mechanical stretch by adding 30 µL to the heart in 1 µL increments as above. Similar to our findings in beating hearts, non-contracting mdx hearts showed increased whole-organ compliance compared to WT, as shown by a blunted increase in LVEDP as LV volume increased (Figure 2c). This is alternatively quantified in Figure 2d as the volume required to raise LVEDP to a given value. Statistical analysis by two-way ANOVA showed significant main effects for strain, volume, and an interaction of the two, indicating that mdx hearts require significantly greater volume to raise LVEDP to 60, 80, 100, and 120 mmHg (Figure 2d). The congruence of our findings in contracting and non-contracting hearts suggests that, in contrast to individual myocytes, the altered whole-organ compliance of mdx hearts is not due to calcium-dependent processes such as sarcomeric contraction or trans-sarcolemmal influx of extracellular calcium [15].

**Figure 3:**
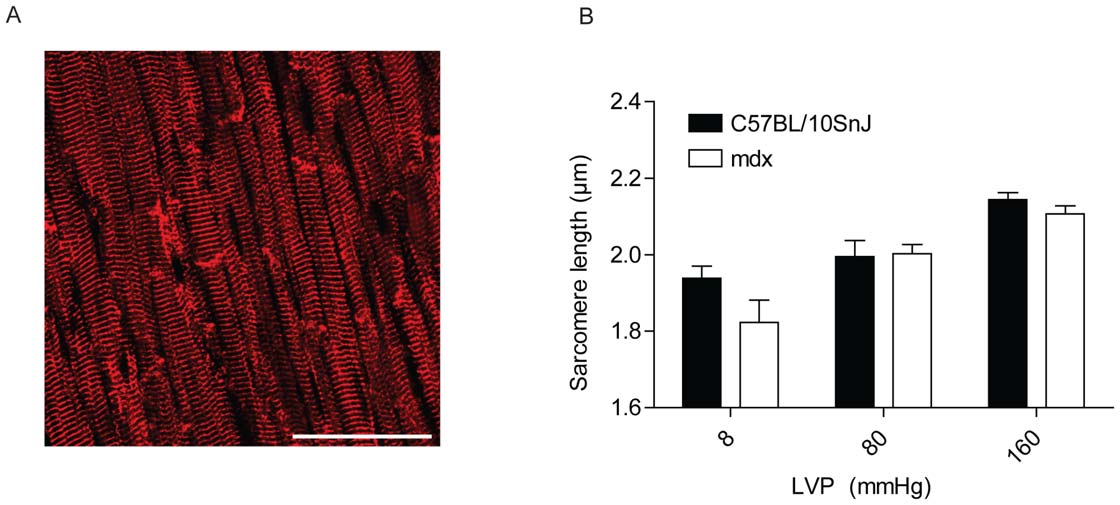
Quantification of changes in sarcomere length during whole-organ stretch in non-contracting hearts. A) Representative image of desmin stained section of fixed heart tissue. Average sarcomere length of circumferentially oriented myocytes from the mid LV free wall was determined. Scale bar = 50 µm. B) Quantitation of average sarcomere length at a range of fixed LV pressures. n = 5–10. No significant differences were detected by two-way ANOVA. Values expressed as mean ± SEM.

The use of non-contracting hearts also allowed for quantitative assessment of end diastolic sarcomere lengths at a set LVEDP. This analysis is confounded in contracting hearts due to obligatory contraction-induced changes in sarcomere length. Accordingly, we measured sarcomere length at a range of set LV pressures to determine the single-cell manifestations of whole-organ stretch in non-contracting WT and mdx hearts. Non-contracting hearts were perfusion-fixed at LVEDP pressures of 8, 80, or 160 mmHg. Immunofluorescent staining for desmin was then used to visualize and measure average sarcomere lengths of the mid-LV free wall (Figure 3a). At each LV pressure tested, mdx and WT hearts had similar sarcomere lengths (Figure 3b). Given that mdx hearts require significantly larger volumes to increase LVP from 8 mmHg to 60, 80, 100, and 120 mmHg (Figure 2d), this suggests altered stretch at the level of individual myocytes did not contribute to increased whole-organ compliance in mdx hearts.

### Whole-organ heart compliance in other genetic models of muscular dystrophy

**Figure 4:**
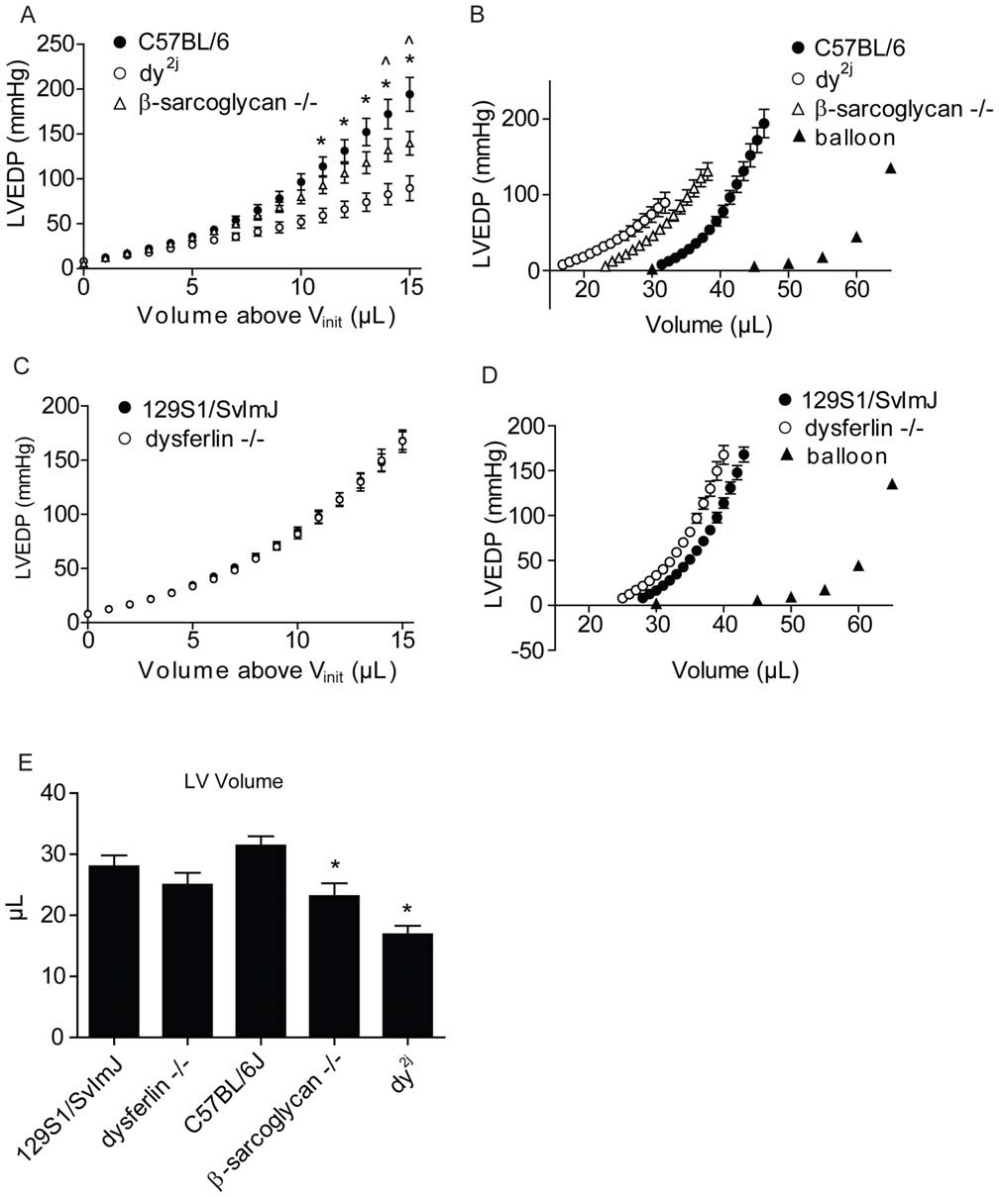
Whole-organ passive compliance of isolated hearts from other models of muscular dystrophy. A) Left, altered compliance of β-sarcoglycan-null and dy^2J^ hearts as shown by plotting LVEDP against volume added above V_init_ during *ex vivo* stretch. B) Estimation of the true LV volume-EDP relationship, plotted as in Figure 1F. C) Left, normal compliance of dysferlin-null hearts as shown by plotting LVEDP against volume added above V_init_ during *ex vivo* stretch. D) Estimation of the true LV volume-EDP relationship. E) Volume required to reach 8 mmHg EDP within the LV (V_init_). n = 5–7. * - p<0.05 for dy^2J^ versus C57BL/6, ∧ - p<0.05 for β-sarcoglycan-null versus C57BL/6. Statistical comparisons made by two-way ANOVA with main effects for strain, volume, and an interaction of the two. Values expressed as mean ± SEM.

Based on the findings above, we hypothesized that increased whole-organ compliance observed in mdx hearts is due to disruption of DGC-mediated connectivity between the extracellular matrix and the intracellular architecture of the cell. To test this hypothesis, we determined the whole-organ compliance of other mouse models of muscular dystrophy in which the DGC or its binding partners are disrupted: β-sarcoglycan-null mice and dy^2j^ mice. β-sarcoglycan is a DGC protein normally expressed in the heart, the lack of which causes Limb-Girdle Muscular Dystrophy Type 2E [44], [45]. Dy^2j^ mice carry a mutation in the LAMA2 gene which causes abnormal splicing of the laminin-α_2_ transcript and expression of a truncated protein [46]. The α_2_ heavy chain subunit of the extracellular matrix protein laminin is bound by α-dystroglycan, facilitating the interaction of the DGC with the extracellular matrix. [47]. Mutations in LAMA2 cause merosin-deficient muscular dystrophy [48]. Similar to mdx mice, the hearts of both β-sarcoglycan null and dy^2j^ hearts showed reduced LV volumes at normal LVEDP and increased whole-organ compliance compared to WT (C57BL/6) hearts (Figure 4a, b, e). Here, similar to mdx mice, we observed increased compliance despite smaller V_init_ for β-sarcoglycan null and dy^2j^ hearts (Figure 4). To determine if the observed effects on compliance in mdx, dy^2j^, and β-sarcoglycan-null hearts are due to specific effects on the DGC or other effects of membrane dysfunction in muscular dystrophy, we also performed the stretch protocol on dysferlin-null hearts. Dysferlin is a membrane repair protein which is not associated with the DGC, the lack of which causes Limb Girdle Muscular Dystrophy Type 2B [49]. These mice have no defect in expression of DGC proteins [50]. Results showed no differences in LV volumes or whole-organ compliance of dysferlin-null and WT control (129S1/SvImJ) hearts (Figure 4c, d, e). Collectively, the findings of altered whole-organ compliance in β-sarcoglycan null and dy^2J^ hearts, but not in dysferlin-null hearts, support the hypothesis that specific disruption of the cytoskeleton-DGC-extracellular matrix alters the mechanical properties of the myocardium and increases whole-organ compliance.

## Discussion

The main new finding of this study is that whole-organ compliance is increased in mdx mice and in other genetic models of muscular dystrophy involving DGC disruption, but not in a model of dystrophy caused by defective membrane repair. These results are evidence that the DGC is an important determinant of organ-level mechanical compliance in the heart. This, in conjunction with our findings of similar changes in sarcomere length despite significantly greater excursions in LV volume lead us to speculate that side-to-side translation of myocytes past each other (myocyte slippage) accounts for the increased compliance observed in mdx, dy^2J^ and β-sarcoglycan-null hearts.

Numerous studies have shown that loss of dystrophin alters the response of striated muscle to mechanical stress. In skeletal muscle, loss of dystrophin alters passive mechanical properties and increases susceptibility to mechanical injury [51], [52], [53]. In cardiac tissue, we and others have previously shown that dystrophin deficient cardiac myocytes are highly susceptible to mechanical injury and show increased passive stiffness [14], [15], [16]. Here, we have subjected isolated hearts to mechanical distension to determine the effects of dystrophin loss on whole-organ compliance and susceptibility to mechanical damage. In agreement with previous findings, we found that mdx hearts show reduced LV volumes compared to controls (Figure 1c) [15]. In contrast to individual cardiac myocytes, isolated mdx hearts show increased compliance compared to normal tissue as LV chamber volume is increased. This is shown in Figure 1f, with the initially left-shifted mdx pressure-volume curve approaches controls as LV volume in increased. This defect is exacerbated in dystrophin/utrophin double knockout hearts and fully prevented in mdx carrier females expressing dystrophin in only 50% of cardiac myocytes. During the distension protocol mdx hearts undergo significant expansion of the LV with no significant effects on cardiac function, membrane damage or cell death. Given the stiffness and mechanical fragility of individual mdx cardiac myocytes during passive stretch [14], [15], this suggests that factors other than mechanical instability of the membrane contribute to the increased whole-organ compliance of mdx isolated hearts. This is further supported by the finding that the altered compliance of mdx hearts was not corrected by P188. In a previous report, Wilding and colleagues performed *ex vivo* stretch and showed no difference between isolated mdx and WT hearts in LV volumes as LVEDP was increased up to 30 mmHg [54]. Our findings are in agreement with this study since we show no significant difference in LVEDP between mdx and WT as volume is increased within this range (Figure 1d). However, here we have conducted a more extensive analysis of the pressure-volume relationship to reveal significant differences in compliance of mdx hearts compared to WT.

To determine possible mechanisms for the observed changes in the mechanical properties of isolated hearts, perfusate calcium concentrations were altered. Increasing, decreasing, or removing calcium altogether did not alter the enhanced compliance mdx hearts. This is evidence that neither trans-sarcolemmal calcium entry through membrane “micro-tears” nor sarcomeric contraction contributes to the increased compliance of mdx hearts. However, we cannot exclude that alterations in expression or activity of other proteins contributes to the increased compliance observed in dystrophic hearts. For instance, caveolin-3 is upregulated in mdx mice [55], interacts with the DGC and numerous cell signaling molecules [56], [57], and participates in buffering mechanical membrane stress [58], and may contribute to the altered mechanical properties of the mdx myocardium.

**Figure 5:**
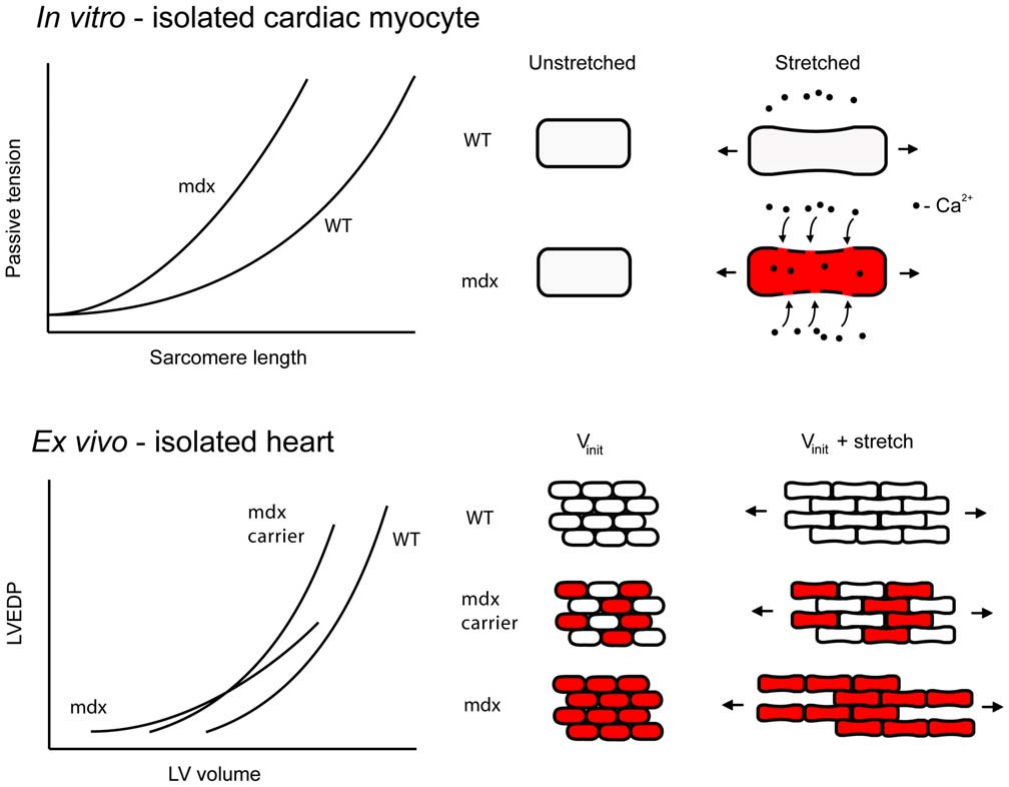
Simplified model for the mechanical properties of the dystrophic myocardium. Top, model for the increased stiffness of mdx myocytes. Stretch causes membrane permeability and influx of extracellular calcium, leading to unregulated contraction (indicated in red). Bottom, simplified model for the effects of dystrophin expression on myocardial compliance. In mdx hearts, membrane permeability may initially cause contraction of LV volume at normal LVEDP, resulting in a left shift in the LV volume-EDP curve. As volume is increased, loss of dystrophin leads to increased slippage of myocytes past one another, leading to increased compliance. This is corrected by expression of dystrophin in 50% of myocytes in mdx carrier females.

To determine the effects of whole-organ stretch on individual myocytes in WT and mdx hearts, we determined sarcomere length at a given LV pressure in non-contracting hearts. Sarcomere lengths were similar at all LV pressures tested despite the considerably larger volumes required to alter LV volume in mdx hearts, suggesting that enhanced stretch of the myocytes does not account for increased compliance in isolated mdx hearts. Since alterations in LV volume expansion are not strictly tracked by proportional changes in sarcomere length, this indicates the mechanical properties of the dystrophic myocardium are not a simple extrapolation of findings from isolated cells. To explain these findings, we propose a model for the accelerated pathological dilation of the ventricle in DMD where disruption of the DGC promotes side-to-side myocyte slippage during whole-organ stretch. A simplified model for this mechanism is shown in Figure 5. Specifically, we propose that as isolated mdx hearts are stretched, loss of dystrophin and disruption of the DGC causes myocytes to translate past each other to a greater degree than occurs in normal hearts. We speculate that, over time, this enhanced myocyte slippage contribute to the pathological dilation of the heart observed in DMD and other forms of inherited muscular dystrophy which involve disruption of cell-extracellular matrix linkages. Therefore, in this model, enhanced compliance in mdx isolated hearts is primarily due to disruptions in the cytoskeleton-DGC-extracellular matrix axis. Additional experiments showed similarly enhanced compliance in β-sarcoglycan-null and dy^2J^ hearts, but not in dysferlin-null hearts, consistent with our hypothesis that disruption of the DGC-mediated linkage of cells with the extracellular matrix promotes enhanced whole-organ cardiac compliance. Collectively, these findings support a mechanism for the increased whole-organ compliance of mdx isolated hearts which is independent of sarcolemma instability and single myocyte compliance. In the broader context of pathophysiological processes associated in DMD cardiomyopathy, the findings presented here suggest a novel mechanism for the pathological ventricular dilation that occurs in DMD and highlight the complex physiological interactions that should be considered when studying diseases at the whole-organ level.

When formulating the working model for the increase compliance of dystrophic hearts observed here, we have also considered other possible explanations for our findings. Specifically, previous reports have shown that chronic ventricular dilation is associated with alterations in the collagen I/collagen III content, promoting a more compliant ventricular wall [59], [60], [61]. However, the tight-skin mouse which expresses a ∼20% excess of collagen in the heart show no abnormalities in passive ventricular compliance [62] and mdx mice typically show no significant deposition of collagen within the myocardium at the ages tested in this study [63]. Additionally, the sarcomeric protein titin has previously been shown to be truncated in skeletal muscle of DMD patients [42], [64], [65]. However, our lab has previously shown that permeabilized cardiac myocytes from the golden retriever muscular dystrophy dog show no difference in passive compliance over a wide range of sarcomere lengths [14]. Previous reports have highlighted the complex arrangement of myocytes in the LV, the anisotropic mechanical properties of the myocardium, and how layers of cardiac myocytes may shear past one another during stretch [66], [67], [68]. Although our model is consistent with the data reported, we cannot exclude that the observed increase in compliance of dystrophic hearts is due to myocardial tissue-level rearrangement, perhaps through shear along myocardial cleavage planes, or altered myocyte orientation within the LV. Additionally, we as show in Figures 1 and 4, the initial LV volumes varied between strains, with some dystrophic strains showing significantly smaller LV volumes compared to controls. In this study we have assumed that the observed differences in LV geometry will not significantly affect our measurement of compliance.

Although LV dilation is associated with numerous disease states of the heart, the basis for this pathological change in organ structure is not well understood. Previous studies suggest that myocyte slippage contributes to LV dilation in numerous disease states including aortic stenosis, acute and chronic myocardial infarction, and end-stage dilated cardiomyopathy [32], [69], [70], [71], [72], [73]. Specifically, these studies have shown that pathological dilation of the LV is associated with LV free wall thinning and reductions in transmural cell number which are not accounted for by hypertrophy, myocyte lengthening or fibrosis [32], [69], [70], [71], [73]. Ultrastructural analysis of the chronically dilated LV has shown that pathological LV dilation is associated with disruption of normal sarcolemmal registry in a dog model of DCM, suggesting myocyte slippage [72]. These findings, along with other reports of disrupted DGC expression in cardiomyopathy brought on by enteroviral infection [74], [75], ischemia [76], [77], acute isoproterenol toxicity [78], or in idiopathic dilated cardiomyopathy [76], [77] lead us to propose that DGC-mediated changes in whole-heart compliance may be a common pathway to pathological LV dilation in numerous forms of cardiac disease. Additionally, the mice used in this study were at an early stage in the progressive cardiomyopathy of mdx mice and typically show minimal evidence of histopathology [63]. Therefore, it is possible that the observed changes in whole-organ compliance in mdx hearts are an initiating event in the remodeling of the dystrophin deficient myocardium. Future studies aimed at determining the age-related changes in cardiac compliance of mdx mice may be useful for understanding the progression of cardiomyopathy in DMD.

With regard to the relevance of the findings presented here to the pathogenesis of heart disease in DMD, there are limitations of this study that need to be considered. The retrograde perfused isolated heart preparation used in this study is a well-established and widely used technique for assessing whole-organ cardiac function as measured during isovolumic contraction and relaxation [79], [80]. However, this technique does not faithfully recapitulate the mechanical rigors of contraction and relaxation due to lack of physiological loading conditions [80]. Previous reports have shown that cardiac function of the dystrophin deficient heart suffers greatly under conditions of physiological loading, due to mechanical instability of the membrane [81], [82]. Although this is certainly a limitation of this study, the absence of physiological loading allowed us to isolate and study mechanical properties of the intact dystrophin deficient heart independent of myocardial damage caused by physiological loading conditions. A significant body of evidence suggests that sarcolemmal instability is the primary cellular defect caused by loss of dystrophin and plays a significant role in pathological remodeling of the heart in DMD [14], [15], [16]. However, we assert that, in addition to the well-established effects of myocyte damage and loss due to mechanical instability of the membrane, the dystrophin deficient heart exhibits a heightened propensity to undergo myocyte slippage which contributes to the progressive pathological dilation of the ventricle in DMD. In addition to this limitation, when using our *ex vivo* stretch protocol we observed differences in whole-organ compliance between WT and mdx hearts at LVEDP well above the normal physiological range. We speculate that the processes resulting in increased compliance in isolated hearts at high LVEDP may also occur under normal conditions *in vivo*, but at a much slower rate. This is in line with the slowly progressive dilation that occurs over the course of years in DMD [26], [27]. Additionally, our model of myocyte slippage at high LVEDP is in agreement with previous findings showing that while the sarcomeric filament and disregulgated calcium handling influences the mechanical properties of the myocardium at lower sarcomere lengths, extracellular matrix proteins contribute significantly at higher sarcomere lengths [15], [83], [84]. Increased compliance in dystrophic hearts was only seen at increased sarcomere lengths, suggesting that these defects were cause by altered extracellular matrix interactions.

In addition to our findings in mdx hearts, we also found similar disturbances in the whole-organ mechanical properties of β-sarcoglycan-null and dy^2J^ hearts. Mutations in β-sarcoglycan and laminin-α_2_ cause muscular dystrophy and, importantly, dilated cardiomyopathy [46], [85], [86], [87], [88]. However, there is evidence from previous studies in skeletal muscle to suggest divergence in the mechanisms by which these mutations cause disease when compared to mdx mice. β-sarcoglycan-null mice show muscle wasting and increased membrane permeability [85]. It is not known if β-sarcoglycan-null mice show enhanced susceptibility to contraction-induced injury, but previous studies have shown significant divergence in susceptibility to contraction-induced injury in genetic models of sarcoglycan deficiency [89], [90], [91]. Dy^2J^ mice show muscle wasting, but no evidence of membrane permeability or susceptibility to contraction-induced injury [92], [93]. Expression of the anti-apoptotic protein BCL2 corrects muscle defects in laminin deficient mice, but not mdx mice, suggesting that aberrant apoptosis plays a role in the pathology of these mice [94]. Despite these differences in the mechanisms of muscle wasting in mdx, dy^2J^, and β-sarcoglycan-null mice, each of these models shows enhanced whole-heart compliance. We speculate that these mouse models share the phenotype of increased whole-heart compliance due to disruptions of cell-extracellular matrix interactions. Genetic ablation of β-sarcoglycan weakens the interaction of α-dystroglycan with the DGC [88] and the mutant laminin-α_2_ heavy chain expressed by dy^2J^ mice is defective in its ability to form stable polymers [95], suggesting mechanisms for the disruption of the contacts between myocytes and the basement membrane. Collectively, the finding of altered mechanical properties in the hearts of mdx, β-sarcoglycan-null and dy^2J^ mice are evidence that altering cell-extracellular matrix contacts is a common mechanism for pathological dilation of the heart in diseases which alter cell-extracellular matrix interactions by a variety of different mechanisms. The lack of mechanical defects in the hearts of dysferlin-null mice further supports this hypothesis. Mutations in dysferlin cause muscular dystrophy, but development of dilated cardiomyopathy is rare [96], [97]. Since dysferlin is not known to directly participate in cell-cell or cell-matrix contacts, the finding of normal compliance in dysferlin-null hearts supports the hypothesis that changes in whole-organ mechanical compliance are specifically caused by disruptions in the cell-extracellular matrix axis of connectivity. The findings presented in this study may therefore have relevance for other disease processes which involve pathological cardiac dilation.

## Materials and Methods

### Mice

C57BL/10SnJ (000666), C57BL/10ScSnJ-DMD^mdx^/J (001801),129-*Dysf^tm1Kcam^*/J (006830), 129S1/SvImJ (002448), B6.WK-*Lama2^dy-2J^*/J (000524), B6.129-*Sgcb^tm1Kcam^*/1J (006832) were purchased from the Jackson Labs (Bar Harbor, Maine). Mdx carrier females were created by breeding C57BL/10SnJ and C57BL/10ScSnJ-DMD^mdx^/J mice. Dystrophin-utrophin double knockout (DKO) mice were a kind gift from Dr. Dawn Lowe at the University of Minnesota [39]. Mice were used at 8–10 weeks of age, with the exception of DKO mice, which were used at 4 weeks due to their severely shortened lifespan.

### Isolated heart preparation

Mice were anaesthetized with sodium pentobarbital and hearts removed via thoracotomy. Hearts were then immediately placed in ice cold modified Krebs-Henseleit buffer (118 mM NaCl, 4.7 mM KCl, 1.2 mM MgSO_4_, 1.2 mM KH_2_PO_4_, 10 mM glucose, 25 mM NaHCO_3_, 2.5 mM CaCl_2_, 0.5 mM EDTA, 2 mM sodium pyruvate) while the aorta was isolated and cannulated. Hearts were then retrograde-perfused through with warm buffer bubbled with 95% O_2_ and 5% CO_2_ to maintain a pH of 7.4. For experiments in non-contracting hearts, isolated hearts were perfused with a similar buffer lacking calcium and including the contractile inhibitor 10 mM 2,3–butanedione monoxime (BDM).The left atrium was then removed and a plastic balloon was inserted into the LV. An in-line pressure transducer allowed for the recording of pressures within the LV. To assess the mechanical compliance of isolated hearts, balloons were inflated to a physiological left ventricular end diastolic pressure (LVEDP) of 8 mmHg. Volume required to bring LVEDP to this normal range was recorded as V_init_.

### *Ex vivo* stretch protocol

To mechanically stretch isolated hearts, volume was added in 1 µL increments. For contracting hearts, 15 µL was added above V_init_. For non-contracting hearts, 30 µL was added above V_init_. In both contracting and non-contracting hearts, the *ex vivo* stretch protocol was performed twice: once to condition the heart and a second time to record functional data. All data reported in this study was taken from the second run through the stretch protocol. Additionally, for this study it was necessary to create balloons with significantly larger volumes than the murine LV to ensure that pressure developed in the balloon was due to pressure against the LV wall and not against the balloon itself.

### Determination of sarcomere length

To determine sarcomere length at given LV pressures, balloons were inflated and clamped to hold LVP at 8, 80 or 160 mmHg in non-contracting hearts, which were then perfused with 10% zinc-formallin and fixed overnight at 4 degrees Celsius. The following day, the LV free wall was dissected, cut transversely at the mid-wall and embedded in paraffin. Serial 5 µm sections were then cut and mounted on histological slides. Immunofluorescence for the Z-disk intermediate filament desmin was then performed to visualize sarcomere length. In brief, sections were dewaxed and rehydrated and blocked with 20% normal goat serum for 1 hour at room temperature. Rabbit anti-desmin primary antibody (Novus Biologicals, NB120-15200) was applied at a 1∶300 dilution for 1 hour at room temperature. After washing, goat anti-rabbit IgG-AlexaFluor 594 (Molecular Probes, A-11037) was applied for 1 hour at room temperature. Sections were then washed and mounted with Vectashield for visualization. Slides were visualized using a Zeiss LSM510 META confocal microscope (Carl Zeiss).

### Lactate dehydrogenase activity assay

Perfusates were collected from isolated hearts immediately before or after the *ex vivo* stretch protocol and immediately frozen in liquid nitrogen. 5 µL of perfusate was then added to 200 µL of solution containing 0.12 mM NADH, 2.3 mM pyruvate, and 0.035% BSA in 100 mM sodium phosphate pH = 7.5. Absorbance at 340 nm was then measured in a plate reader (FLUOstar Omega, BMG Labtech) at 1 minute intervals for 15 minutes at a constant temperature of 37 degrees Celsius.

### Statistics

Comparisons between more than 2 groups were made by one-way analysis of variance with a Tukey post-test. When more than one independent variable was tested, two-way analysis of variance with a Bonferroni post-test was used to compare groups. All statistical analysis was carried out using Prism (GraphPad Software).

## Supporting Information

Figure S1 **P188 does not affect whole-organ compliance and e** ***x*** **vivo stretch does not cause ischemia or impair cardiac function.** A) Whole-organ compliance of isolated mdx hearts with and without poloxamer 188 in the perfusate (mdx data is same data used in Figure 1). B) V_init_ of mdx perfused with and without poloxamer 188 perfusate (mdx data is same data used in Figure 1). C) Rate of perfusate flow through the coronary vasculature in mdx and WT hearts during *ex vivo* stretch protocol. D) LDH activity in perfusates from WT and mdx hearts taken before and after *ex vivo* stretch protocol. E) Comparison of LVDP before and after *ex vivo* stretch protocol. No significant differences between observed between WT and mdx mice by two-way ANOVA. For A, B, n = 7–14. For C, n = 11–14. For D, n = 5–9. For E, n = 8–12.* - p<0.05 vs. all other groups by one-way ANOVA. Values expressed as mean ± SEM.
(TIF)

Table S1 **Baseline** ***ex vivo*** **functional data for isolated WT and mdx hearts.** Contractile function as shown by LV developed pressure (LVDP) and the maximum derivatives of LV pressure (dP/dt max.) were similar between groups. Lusitropic function as measured by LV end diastolic pressure (LVEDP) and the minimum derivative of LV pressure (dP/dt min.) were also similar between the groups by student's t-test. n = 12–15. Values expressed as mean ± SEM.
(TIF)
