# Supplementary figures and images for: Ex Vivo Stretch Reveals Altered Mechanical Properties of Isolated Dystrophin-Deficient Hearts
Source: PLoS One. 2012 Mar 9;7(3):e32880. doi: 10.1371/journal.pone.0032880 (PMC3298453; doi:10.1371/journal.pone.0032880)

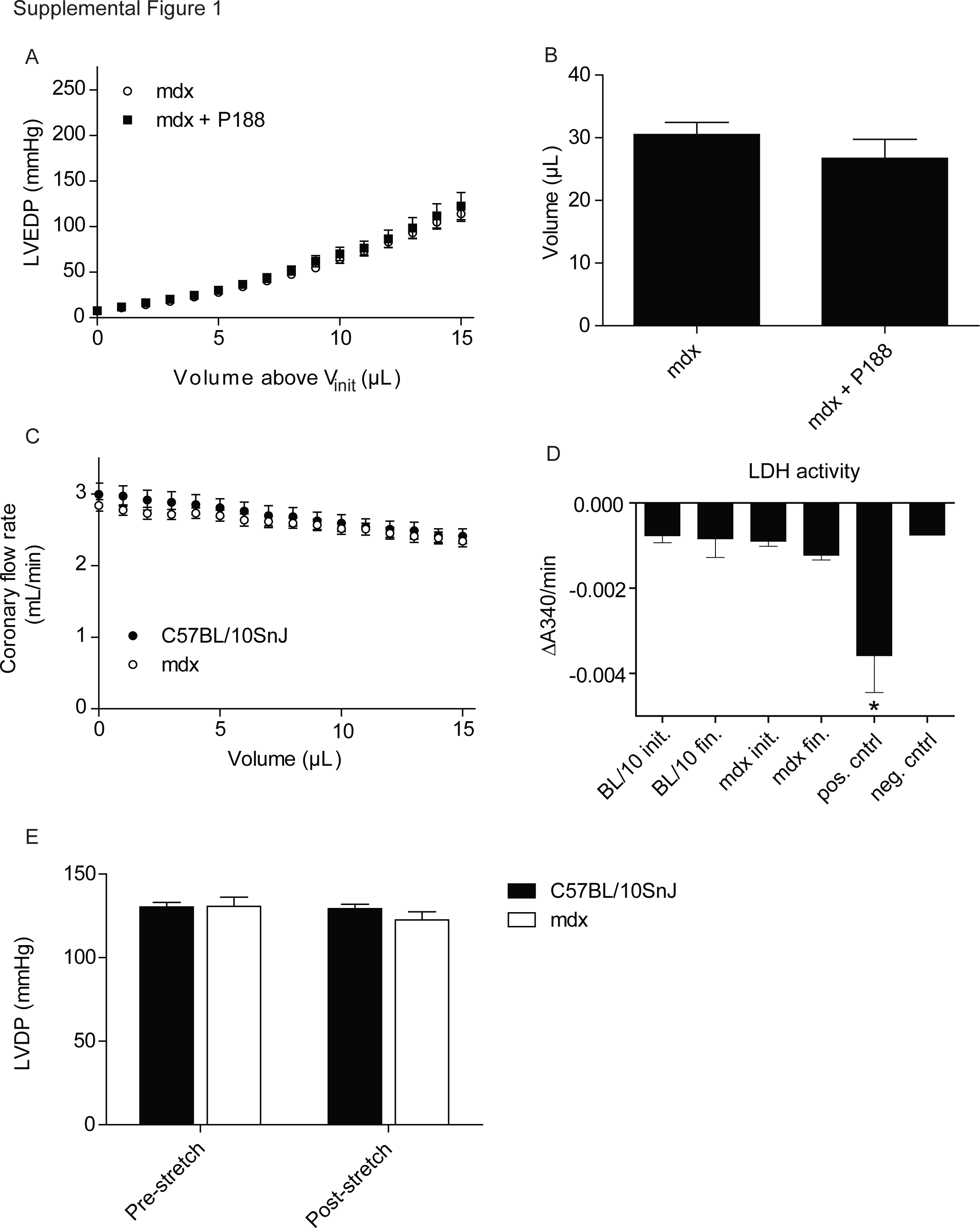

Supplement: Figure S1 — P188 does not affect whole-organ compliance and e x vivo stretch does not cause ischemia or impair cardiac function. A) Whole-organ compliance of isolated mdx hearts with and without poloxamer 188 in the perfusate (mdx data is same data used in Figure 1). B) Vinit of mdx perfused with and without poloxamer 188 perfusate (mdx data is same data used in Figure 1). C) Rate of perfusate flow through the coronary vasculature in mdx and WT hearts during ex vivo stretch protocol. D) LDH activity in perfusates from WT and mdx hearts taken before and after ex vivo stretch protocol. E) Comparison of LVDP before and after ex vivo stretch protocol. No significant differences between observed between WT and mdx mice by two-way ANOVA. For A, B, n = 7–14. For C, n = 11–14. For D, n = 5–9. For E, n = 8–12.* - p<0.05 vs. all other groups by one-way ANOVA. Values expressed as mean ± SEM. (TIF) [file pone.0032880.s001.tif]

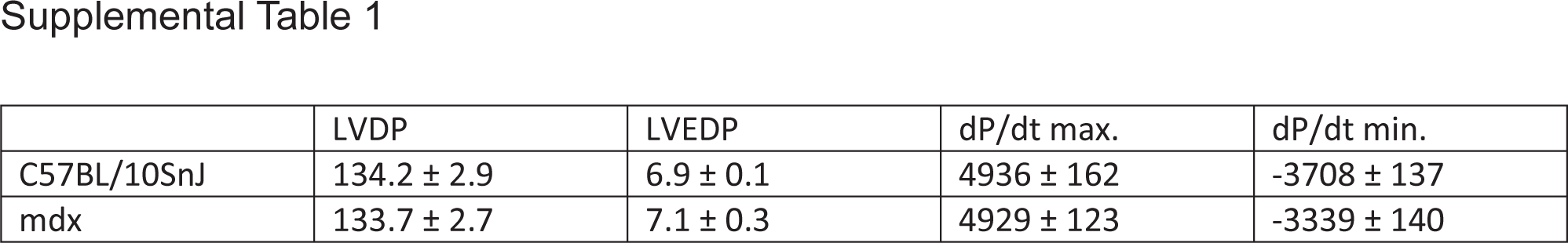

Supplement: Table S1 — Baseline ex vivo functional data for isolated WT and mdx hearts. Contractile function as shown by LV developed pressure (LVDP) and the maximum derivatives of LV pressure (dP/dt max.) were similar between groups. Lusitropic function as measured by LV end diastolic pressure (LVEDP) and the minimum derivative of LV pressure (dP/dt min.) were also similar between the groups by student's t-test. n = 12–15. Values expressed as mean ± SEM. (TIF) [file pone.0032880.s002.tif]
